# The Indirect Benefits of Mating with Attractive Males Outweigh the Direct Costs

**DOI:** 10.1371/journal.pbio.0030033

**Published:** 2005-01-25

**Authors:** Megan L Head, John Hunt, Michael D Jennions, Robert Brooks

**Affiliations:** **1**School of Biological, Earth and Environmental Sciencesthe University of New South Wales, SydneyAustralia; **2**School of Botany and Zoology, The Australian National UniversityCanberraAustralia; University of OxfordUnited Kingdom

## Abstract

The fitness consequences of mate choice are a source of ongoing debate in evolutionary biology. Recent theory predicts that indirect benefits of female choice due to offspring inheriting superior genes are likely to be negated when there are direct costs associated with choice, including any costs of mating with attractive males. To estimate the fitness consequences of mating with males of varying attractiveness, we housed female house crickets, Acheta domesticus, with either attractive or unattractive males and measured a variety of direct and indirect fitness components. These fitness components were combined to give relative estimates of the number of grandchildren produced and the intrinsic rate of increase (relative net fitness). We found that females mated to attractive males incur a substantial survival cost. However, these costs are cancelled out and may be outweighed by the benefits of having offspring with elevated fitness. This benefit is due predominantly, but not exclusively, to the effect of an increase in sons' attractiveness. Our results suggest that the direct costs that females experience when mating with attractive males can be outweighed by indirect benefits. They also reveal the value of estimating the net fitness consequences of a mating strategy by including measures of offspring quality in estimates of fitness.

## Abstract 2

Experiments reveal that female crickets that choose an attractive mate have lower survival, but that this can be cancelled out or even outweighed by the increased fitness of her offspring

## Introduction

Whether mate choice can be maintained by indirect selection when females incur direct costs by being choosy is the subject of ongoing theoretical controversy [1,2,3,4,5]. This is particularly true when the principal or only benefit of mating with attractive males is that they sire attractive sons. Weatherhead and Robertson [6] suggested 25 y ago that the genetic benefits of mating with an attractive male could outweigh the cost of reduced investment in parental care that such a male makes. This suggestion has been opposed by several important theoretical models [5,7,8]. More generally, some recent theoretical work has suggested that because of the weakness of indirect selection relative to direct selection, genetic benefits of choice are likely to have little effect on the evolution of costly mate choice [2,3]. This assertion has been contested by other theoretical work [1].

In order to understand how mate choice evolves, it is necessary to estimate the overall effect of mate choice on female fitness [9,10,11,12]. The effect of mating with males of differing attractiveness on total female fitness depends on both positive and negative effects on a variety of fitness components. The signs and strengths of these effects are paramount to distinguishing between the relative importance of various models of mate-choice evolution.

Evidence from studies that have measured one or a few fitness components has been invoked to support direct benefits [13,14], “viability genes” [15,16,17,18], “Fisherian runaway” [19,20], and “sexually antagonistic coevolution” [21,22] models of mate-choice evolution. Similar evidence has also been used in tests for differential allocation of reproductive effort to offspring sired by attractive males [14,23,24]. Understanding the relative significance of these processes, however, requires measuring as complete a set of fitness components as possible [12,19] and estimation of the multigenerational effects of mate choice on fitness [11,25] through both sons and daughters [12,26]. To date, only two studies have compared the number of grandchildren produced when females mate with attractive or unattractive males [10,16]. Unfortunately, neither study accounted for the beneficial effects of heritable male attractiveness, an important consideration in most models of mate-choice evolution.

How fitness should be estimated is controversial [27,28]. Measuring total fitness is logistically preclusive, but rate-insensitive estimates, such as the number of grandchildren, or rate-sensitive estimates, such as the intrinsic rate of increase, may offer reasonable approximations [10,11,25]. The key difference between these two estimates is that rate-sensitive estimates take into account both the timing of reproduction and the developmental time of offspring, whereas rate-insensitive measures do not. To date, most empirical studies have employed rate-insensitive estimates, whereas theoretical models tend to focus on the intrinsic rate of increase [28].

Here, we measured both direct and indirect fitness components of female house crickets, Acheta domesticus, mated to either attractive or unattractive males for the term of their adult life span. We present a female's total fitness as both a rate-sensitive (the intrinsic rate of increase) and a rate-insensitive estimate of fitness (the total number of grandchildren) in interpreting our findings.

## Results

Our treatment did not affect the number of grandchildren produced via daughters, via sons, or in total (Table 1). Thus there was no difference in the rate-insensitive estimate of fitness for females mated to males of differing attractiveness. Females that mated with attractive males did, however, experience higher relative intrinsic rates of increase (*r_est_*) than females mated with unattractive males (Table 2).

**Table 1:**
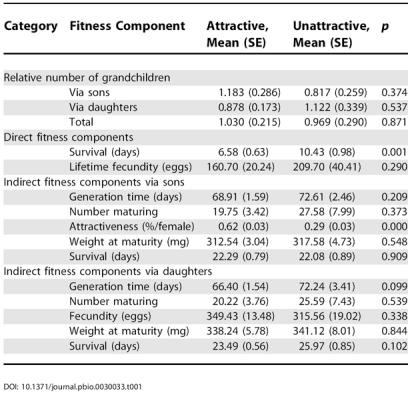
The Effects of Mating with Either Attractive or Unattractive Males on a Number of Fitness Components

**Table 2:**
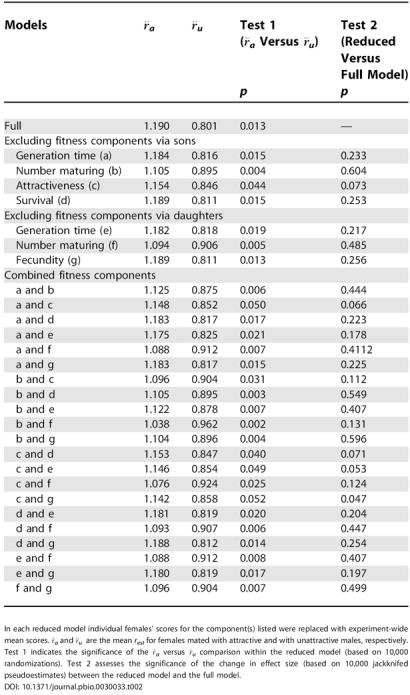
The Sensitivity of *r_est_* to Variation in Individual and Combined Fitness Components

In each reduced model individual females' scores for the component(s) listed were replaced with experiment-wide mean scores. *r¯_a_* and *r¯_u_* are the mean *r_est_* for females mated with attractive and with unattractive males, respectively. Test 1 indicates the significance of the *r¯_a_* versus *r¯_u_* comparison within the reduced model (based on 10,000 randomizations). Test 2 assesses the significance of the change in effect size (based on 10,000 jackknifed pseudoestimates) between the reduced model and the full model

**Figure 1:**
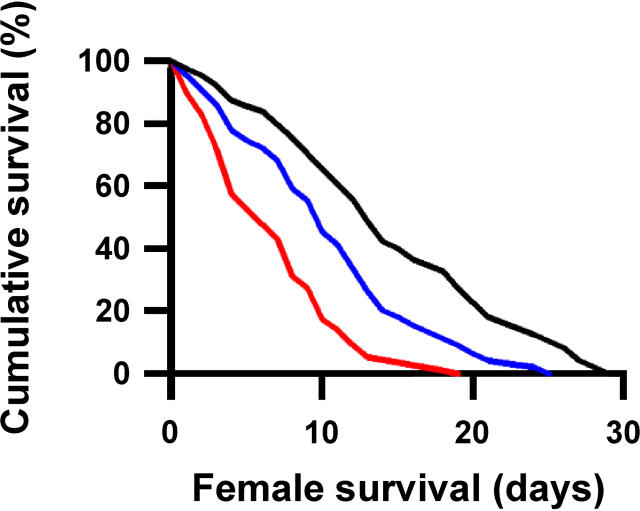
Female Survival in Relation to Experimental Treatment Females housed alone (black line) survived longer than females housed with either type of male (Cox regression Wald_1_ = 29.636, *p* = 0.000). Females mated to unattractive males (blue line) survived longer than females mated to attractive males (red line) (Wald_1_ = 10.802, *p* = 0.001) *n* = 40, 40, 40.

The overall difference between the treatments on *r_est_* was not due to any single fitness component (Table 2). When looking at the fitness components individually, the strongest effects were a survival cost experienced by females mated to attractive males (Figure 1), and an indirect benefit because sons of attractive males were more than twice as likely to mate as those of unattractive males (see Table 1). However, neither of these components alone can explain the significant difference in *r_est_* between females mated to attractive or to unattractive males (see Table 2). Treatment differences in other fitness components, although individually not significant, still influenced our estimates of the overall fitness consequences of mating with attractive males. In particular the combined effect of sons' attractiveness and daughters' fecundity had a significant effect on our model (see Table 2).

When we combined a female's egg number, egg width, and egg length (from the first week of egg laying) into a single index of reproductive effort, we found that females mated to attractive males exerted greater reproductive effort in the first week of the experiment than those mated to unattractive males (principal component 1: attractive = 0.239 ± 0.116, unattractive = −0.233 ± 0.199, randomisation test *p* = 0.043). Of the constituent measures of week 1 reproductive effort, only egg width differed significantly between treatments (egg number: attractive = 129.07 ± 15.08, unattractive = 108.17 ± 18.84, *p* = 0.382; egg width: attractive = 0.618 ± 0.008, unattractive = 0.568 ± 0.014, *p* = 0.005; egg length: attractive = 2.71 ± 0.017, unattractive = 2.68 ± 0.025, *p* = 0.373).

## Discussion

To provide an inclusive estimate of the total fitness consequences of mating with an attractive or unattractive male, we quantified both the direct costs to females and the indirect benefits to their offspring. We made two main findings. First, the mating-associated costs borne by females are greater when mating to attractive males throughout their life than when they are mated to unattractive males. Second, these costs are cancelled out (when we use the rate-insensitive measure of the number of grandchildren) and may be outweighed by (when we use the rate-sensitive estimate of the intrinsic rate of increase) the benefits of having offspring with elevated fitness (i.e., indirect benefits).

Contrary to some theoretical predictions [2,3,5], but see [1,6], our results suggest that it may be possible for female mate choice to evolve via indirect benefits, despite the presence of direct costs. Whether this is the case or not will, however, depend on the magnitude of other costs of choice not measured here, such as the costs associated with being choosy, as well as the accuracy of female choice [3].

The costs of choice, including the costs of mating with attractive males, are of central importance to theoretic models of mate-choice evolution [1,2,3,4,5,29]. In many species females incur survival or fecundity costs due to being courted or harassed by males [30,31], mating [32,33], and allocating resources to egg laying, gestation, and/or parental care [34]. Female Drosophila melanogaster mated to large (and thus presumably attractive) males incur a greater survival cost than females mated to smaller males [21,22], and this appears to be due to a higher mating rate with large males [22]. A potential criticism of such studies is that they are based on single traits that are taken to be an indirect measure of a male's attractiveness. By using a direct biological measure that incorporates all traits that contribute to a male's ability to induce a female to mate during short-range courtship, our results provide the first direct evidence that females sustain greater direct costs when mating with males that are more attractive in this context.

While we do not know the exact mechanisms driving the survival cost seen in our experiment, our finding that females mated to more attractive males experience lower survival is consistent with sexual conflict between males and females over mating decisions [25,35], and with differential allocation [34]. Females mated to attractive males exerted greater reproductive effort in the first week of the experiment. This could be the result of male manipulation, for example, increased mating rate [32], or stimulants in seminal fluids [36,37,38] whereby more attractive males manipulate females to invest more in their offspring than is optimal for the females. The possibility of male manipulation is also supported by a study by Murtaugh and Denlinger [39], which shows that in A. domesticus, males pass substances in their ejaculate that promote higher rates of short-term oviposition. Alternatively, it may be adaptive for females to invest more in the offspring of attractive males [34,40]. Differential allocation is only likely to be adaptive if there is an indirect fitness benefit to allocating greater reproductive effort when mated to attractive males [34]. The indirect fitness benefits that we report here, particularly the benefit of having more attractive sons, may provide an adaptive basis for differential allocation by females to the offspring of more attractive males.

Several studies have reported fitness benefits of mating with attractive males. Females mated to such males have been reported to have offspring that have greater longevity [15,41], faster growth rate [16,17,42], increased fecundity of daughters [16,42], and increased attractiveness of sons [19,20,42,43,44]. In our study, the net fitness benefit of mating with attractive males is not due to any single indirect benefit but to a combination of fitness components. This illustrates the importance of measuring net fitness, especially if fitness components act in opposition to each other.

A number of studies have proposed the use of an aggregate measure of male attractiveness rather than a single morphological indicator [44,45]. Our use of time to mounting allows us to gain a measure of male attractiveness that is based on all traits that contribute to male mating success (hence ‘attractiveness') during short-range courtship interactions [46]. It is the use of such a measure that may explain the high correlation between fathers' and sons' attractiveness in this experiment and others based on similar measures [12,44]. The greater attractiveness of sons sired by attractive males may also be explained by differential allocation; studies have shown that maternal effects may enhance the heritability of male traits [47]. An important role for maternal effects is unlikely in our experiment, however, because no other fitness components of sons or daughters differ significantly between the treatments. Regardless of whether sons' greater attractiveness is due to additive genetic variation for attractiveness per se or to the ability to manipulate females into allocating more resources to the offspring, such a trait will increase a female's net fitness if it increases the reproductive success of her sons sufficiently.

Due to the nature of our experimental design we were unable to measure all the costs and benefits associated with choosing and mating with attractive males. First, we did not measure sons' ability to compete with other males for access to females. However, in this population of A. domesticus, fighting ability has been shown to be positively correlated with attractiveness as we have measured it here [48,49]. Thus, if anything we may have underestimated the fitness benefit gained through having attractive sons. Second, we did not measure long-range attraction of males through advertisement calling. Third, our design simplifies the way mating takes place for females paired with attractive or unattractive males. Pairing females with a single male for 7 d at a time may decrease or increase the costs associated with mating with males. For instance, costs may be increased because females are unable to escape male harassment, or they may be decreased because there is no male–male competition. Despite these limitations, we believe that our estimate of the intrinsic rate of increase offers a reasonable approximation of net fitness.

The fitness estimate of choice in empirical studies may depend on the importance of reproductive timing in the system in question [28]. Brommer et al. [27] compared estimates of lifetime reproductive success and intrinsic rate of increase to real long-term data from two species of bird. They showed that lifetime reproductive success was a better estimate of genetic contribution to future generations. However, their estimates did not include measures of offspring quality, and as they point out, their results may depend on the species life history, and the generality of their conclusions thus remains to be tested.

There are several reasons why reproducing early and having short maturation times is likely to be advantageous in crickets. First, extrinsic mortality of crickets in the wild is likely to be high. Second, females become less choosy [50], lose condition, and produce fewer eggs as they age (M. L. Head, unpublished data). Also, individuals with shorter generation times will contribute their genes to future generations more rapidly [51].

Our research constitutes one of the first attempts to directly and simultaneously test the combined direct and indirect effects of mating with males that differ in attractiveness. Only by following the effects of mating with attractive or unattractive males through at least two generations, and through both sons and daughters, is it possible to observe the combined direct effects on female lifetime fecundity and the genetic effects on offspring fitness [11,12,25]. Although the need to conduct such a study under laboratory conditions may constrain our ability to definitively answer this question, our results suggest that indirect genetic benefits have the potential to outweigh direct costs of mating with attractive males. Moreover, this effect comes about largely, but not exclusively, due to the production of more attractive sons.

## Materials and Methods

### Study species

We obtained approximately 1,000 final-instar A. domesticus nymphs from a commercial cricket breeder (Pisces Enterprises, Phoenix, Arizona, United States). Nymphs were separated into single-sex culture tubs (4 × 80 l containers per sex) to ensure their virginity, and reared with constant access to food (Friskies Go-Cat senior) and water until eclosion. At eclosion, adults were maintained in single-sex cultures for a further 10 d to ensure sexual maturity.

In the cultures from which the insects have been derived, crickets are raised in densities ranging from 23,000–34,000 m^−3^ and fed grain ad libitum. In these conditions males and females mate multiply. Males fight with other males and court females, and there is a positive relationship between male dominance and attractiveness [49]. Despite high densities, female cooperation is needed for mating to occur because a female must actively mount the male and align her genitalia with his to mate. Mate choice in both culture and wild populations of A. domesticus is generally sequential. That is, females choose males by either mating or rejecting males one at a time, rather than choosing between males simultaneously. We chose to work on cultured A. domesticus because our laboratory conditions closely resemble the culture conditions under which they have recently evolved. This similarity maximises the evolutionary relevance of our measures. Our experimental design, however, requires that females be kept alone, creating an important environmental difference from the culture conditions to which females have been adapted.

#### Male attractiveness

The attractiveness trials throughout our experiment were based on latency to mounting for pairs of crickets. While this protocol does not allow all elements of female choice to be measured, in A. domesticus a female mounting a male is a reliable predictor of mating success (in a previous study 46 out of 50 mountings led to successful transfer of a spermatophore [49]). Also, females have been shown to produce more eggs for males that they choose quickly (M. L. Head, unpublished data). This indicates that latency to mounting is representative of other aspects of choice in this species.

To obtain males that were either attractive or unattractive to females we ran a two-round tournament. In round one, each male was placed in a clear plastic container (7 × 7 × 5 cm) with a single randomly assigned female, at night, under red lighting. When a female mounted a male, but before spermatophore transfer, they were separated. Once half of the females had mounted, all remaining pairs were separated. Round two commenced with a new female assigned at random to each male. The first half of first-round mounted males to be remounted became our “attractive” treatment males. The half of first-round unmounted males that remained unmounted longest in round two became the “unattractive” treatment males. Only males that courted females during the tournament were used. This biological assay of male attractiveness incorporates all traits that make a male attractive during short-range courtship, rather than a single trait correlated with attractiveness (see [10,11,44]).

#### Experimental design

Forty females were randomly assigned to each of three treatments: attractive, unattractive, and an unmated control. Females were weighed and placed individually in a small plastic container (as above) with food, water, and a petri dish of moist sand for egg laying. Males from the appropriate treatment were randomly assigned to a female. Every 7 d, or whenever a male died, a new male from the same treatment (but from a new tournament) was placed with the female. This allowed us to measure the fitness consequences of the strategy of mating with attractive or unattractive males, rather than the consequences of mating with a given individual male. Food, water, and sand were replaced every 7 d.

#### Fitness measures

Female survival was monitored daily, and the number of eggs laid was counted weekly. Hatching success was estimated as the proportion of eggs that hatched within 14 d of the first egg hatching in each collection. Hatchlings were collected every 3 d, and their mean weight was recorded. Each week, 50 hatchlings per female were separated into two boxes (20 × 13 × 13 cm), each containing 25 nymphs. We monitored offspring survival every 7 d and recorded the time to mature and sex and body weight at eclosion.

If a female had fewer than 50 hatchlings in a given week these were discarded. For these females, the actual number of hatchlings was multiplied by the overall experimental mean for each subsequent offspring fitness measure, to predict the number of grandchildren produced. This is a conservative approach to missing values because it reduces the difference between the treatments.

Offspring generation times were calculated from the time females were first placed with a male until the offspring matured. This takes into account not only the time it takes for the offspring to mature, but also the timing of egg laying. Mature offspring were housed individually, and their survival monitored daily. Ten days after eclosion each son's attractiveness was estimated by placing him with a stock virgin female in a small plastic container for 90 min. Mounted males were separated from females before spermatophore transfer occurred. We used the proportion of a female's sons that were successful in this assay as our measure of sons' average attractiveness (e.g., if 8 of 16 sons were mounted, we assumed that, on average, each son had a 50% chance of mating per encounter with a female).

Ten days after eclosion daughters were placed with a stock male for 12 h to allow mating. Afterwards, survival of sons and daughters was again monitored daily, and sand was collected from daughters weekly. Eggs from daughters were counted to estimate lifetime fecundity.

#### Statistical analysis

We calculated two estimates of female relative net fitness when mating with either an attractive or unattractive male. A rate-insensitive estimate, the relative number of grandchildren produced by a female (*g_est_*)*,* and a rate-sensitive estimate, *r_est_*.

To estimate the absolute number of grandchildren each female had (*G_est_*)*,* we added the number of grandchildren she had through daughters, estimated as



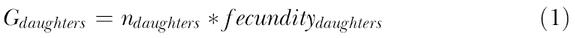



to the number she had through sons, *G_sons_,* estimated as



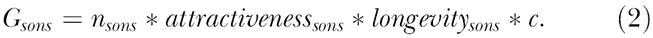



The attractiveness of a female's sons was estimated as the proportion of her sons that were mounted in attractiveness trials; longevity is the mean adult life span of a female's sons and *c* is the ratio of the total number of grandchildren through daughters in the experiment to the total number of sons mounted in the attractiveness trials. This correction factor converts an attractiveness score into units of the number of grandchildren. Using this correction factor ensured that mean son and daughter reproductive success across the entire experiment was equal, satisfying the assumption that mean reproductive success for males and females is the same in populations with an equal sex ratio [52].

*G_est_* for each female was then divided by the experimental mean to give the relative *g_est_.*

We estimated the absolute intrinsic rate of increase for each female as



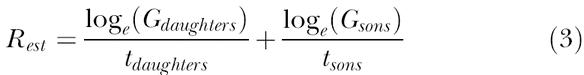



where *t* is the generation time from parental first mating to offspring maturity in a particular lineage. We converted our rate-sensitive measure into a measure of relative intrinsic rate of increase (*r_est_*)*,* by dividing each female's *R_est_* by the experiment-wide mean.

Due to the non-normal distributions of many fitness components, we tested the significance of treatment differences for each fitness component using two-tailed randomisation tests. In each randomisation test the observed data was randomly assigned to the two treatments 10,000 times. *P*-values are based on the proportion of randomisations in which the absolute value of the estimated difference was greater than that observed in the original data.

To explore the sensitivity of our estimates of *r_est_* to variation in each fitness component we used a model-building approach. We removed the variance of each fitness component from our full model, in turn, by assigning every female the overall experimental mean value of that component. We similarly excluded every combination of two fitness components. We then ran a randomization test (as above, 10,000 randomizations) for each reduced model to test whether the treatment effect remained. We also obtained 10,000 jackknifed estimates of the difference between the treatments for each reduced model (by randomly omitting 20% of the sample in each estimate), to test whether the reduced model resulted in a significantly different effect size than the original full model. *P*-values are based on the proportion of jackknifed estimates in which the absolute value of the difference between the treatments was greater than the absolute difference in the full model.

We used principal components analysis to investigate the effects of mating with attractive or unattractive males on week 1 reproductive effort via egg number, egg width, and egg length. All three measures showed a strong positive loading on the first principal component, which explained 66% of the variation in the constituent measures. We then tested for differences in female reproductive effort between the treatments using a randomisation test.

## Abbreviation

*r_est_*: relative intrinsic rate of increase
